# Detection rate of Pompe disease in undiagnosed neuromuscular patients from four major centres in the UK - results of a 12 month prospective audit

**DOI:** 10.1186/1471-2474-14-S2-P20

**Published:** 2013-05-29

**Authors:** Tracey Willis, Mark Roberts, David Hilton-Jones, Ros Quinlivan, Mike Hanna, Volker Straub

**Affiliations:** 1 Robert Jones and Agnes Hunt, Oswestry, UK and Birmingham Children’s Hospital, Birmingham, UK; 2 Salford Royal NHS Foundation Trust, Salford, UK; 3 John Radcliffe Hospital, Department of Clinical Neurology, Oxford, UK; 4 MRC Centre for Neuromuscular Diseases and UCL, Institute of Neurology, London, UK; 5 Institute of Genetic Medicine, Newcastle University, International Centre for Life, Newcastle upon Tyne, UK

## Introduction

This 12-month audit (conducted between 1^st^ July 2011 to 30^th^ June 2012) was designed following a retrospective audit in a specialist neuromuscular centre (UK) using DBS testing to identify late-onset Pompe disease. The objective was to screen all patients with an unknown diagnosis presenting with a limb girdle pattern of muscle weakness +/- respiratory symptoms +/- scoliosis +/- rigid spine that were seen in the clinic, including new referrals and follow-up patients. Our goal is to improve the detection rate of Pompe disease and to initiate early treatment in clinical practice. To that end, this audit has been extended through June 2013.

## Results

Out of 102 patients, six positive tests were obtained on DBS (5.9%), one of which was a false positive in a patient aged 40 years (CK>1000, symptoms from adulthood, resp. problems; ventilated at night). Five positive cases were subsequently confirmed (4.9% - plus one additional case identified as a family member). These cases will be reported in detail. If not in the audit, these patients would not have been diagnosed with Pompe disease on the basis of their symptoms.

## Conclusion

Pompe disease is a progressive, debilitating and often fatal neuromuscular disorder that presents with a continuum of clinical phenotypes that vary with regards to organ involvement, age of onset, disease severity and rate of progression. Early diagnosis is key to implementing early disease modifying treatment. Whilst all these patients detected have some of the features that have been documented in Pompe disease, it is vital that clinicians are alerted to the subtle presentation that late onset Pompe can have. DBS is a rapid test that improves the time to diagnosis, and hence, time to treatment, as suggested in the International Consensus meeting [1].
